# Hidden mechanical weaknesses within lava domes provided by buried high-porosity hydrothermal alteration zones

**DOI:** 10.1038/s41598-022-06765-9

**Published:** 2022-02-25

**Authors:** Herlan Darmawan, Valentin R. Troll, Thomas R. Walter, Frances M. Deegan, Harri Geiger, Michael J. Heap, Nadhirah Seraphine, Chris Harris, Hanik Humaida, Daniel Müller

**Affiliations:** 1 Laboratory of Geophysics, Department of Physics, Faculty of Mathematics and Natural Sciences, Universitas Gadjah Mada, Yogyakarta, Indonesia; 2 Department of Earth Science, Natural Resources and Sustainable Development (NRHU), Uppsala University, Villavägen 16, Uppsala, Sweden; 3 Centre for Natural Hazards and Disaster Science (CNDS), Uppsala University, Villavägen 16, Uppsala, Sweden; 4 Faculty of Geological Engineering, Universitas Padjajaran (UNPAD), Bandung, West Java Indonesia; 5 Department of Geophysics, GFZ German Research Center for Geosciences, Telegrafenberg, Potsdam, Germany; 6 Institute of Earth and Environmental Sciences, University of Freiburg, Albert Str. 23b, Freiburg im Breisgau, Germany; 7 CNRS, Institut Terre et Environnement de Strasbourg, Université de Strasbourg, 5 rue René Descartes, Strasbourg, France; 8 Institut Universitaire de France (IUF), Paris, France; 9 Department of Geological Science, University of Cape Town, Rondebosch, South Africa; 10 BPPTKG (Balai Penyelidikan Dan Pengembangan Teknologi Kebencanaan Geologi), Jalan Cendana 15, Yogyakarta, Indonesia

**Keywords:** Natural hazards, Solid Earth sciences

## Abstract

Catastrophic lava dome collapse is considered an unpredictable volcanic hazard because the physical properties, stress conditions, and internal structure of lava domes are not well understood and can change rapidly through time. To explain the locations of dome instabilities at Merapi volcano, Indonesia, we combined geochemical and mineralogical analyses, rock physical property measurements, drone-based photogrammetry, and geoinformatics. We show that a horseshoe-shaped alteration zone that formed in 2014 was subsequently buried by renewed lava extrusion in 2018. Drone data, as well as geomechanical, mineralogical, and oxygen isotope data suggest that this zone is characterized by high-porosity hydrothermally altered materials that are mechanically weak. We additionally show that the new lava dome is currently collapsing along this now-hidden weak alteration zone, highlighting that a detailed understanding of dome architecture, made possible using the monitoring techniques employed here, is essential for assessing hazards associated with dome and edifice failure at volcanoes worldwide.

## Introduction

Catastrophic volcano dome collapse is a highly hazardous, but poorly understood, phenomenon. Historic and recent global examples of dome or partial dome collapse include Soufrière Hills volcano, Montserrat in 1998–1999^1^, Unzen volcano, Japan in 1991^2^, Mount Hood and Mount Rainier in USA^3,4^, La Soufrière de Guadeloupe in the Eastern Caribbean^5^, Santiaguito in Guatemala^6^, Volcán de Colima in Mexico^7^, and Merapi and Sinabung volcanoes in Indonesia in 2010 and 2013–2014, respectively^8–10^. Crucially, it has been observed that the debris avalanche materials resulting from such dome collapse events frequently contain moderately to highly altered rock clasts, pointing towards a likely crucial role for pre-collapse mechanical weakening of these dome complexes as a result of hydrothermal alteration^11,12^. Indeed, previous studies have highlighted that weakened hydrothermally altered zones can promote volcano instability and collapse^4,13–16^, but the fundamental problem that remains concerning the structural weakening of lava domes and volcano flanks is the difficulty in assessing the exact locations of instability. Here drone surveys may be the only method that can provide sufficient image resolution and flexibility to help interpret destabilizing lava domes and their morphology^17–19^. As direct rock sampling and analysis is frequently challenging at active lava domes, there is a need for a more integrated (cross-method) approach for improving our understanding of the structural, chemical, and mineralogical processes at work. This is especially true with respect to buried (or hidden) zones of structural weaknesses which, if addressed, could help to substantially advance our predictive capability of catastrophic dome failures.

**Figure 1:**
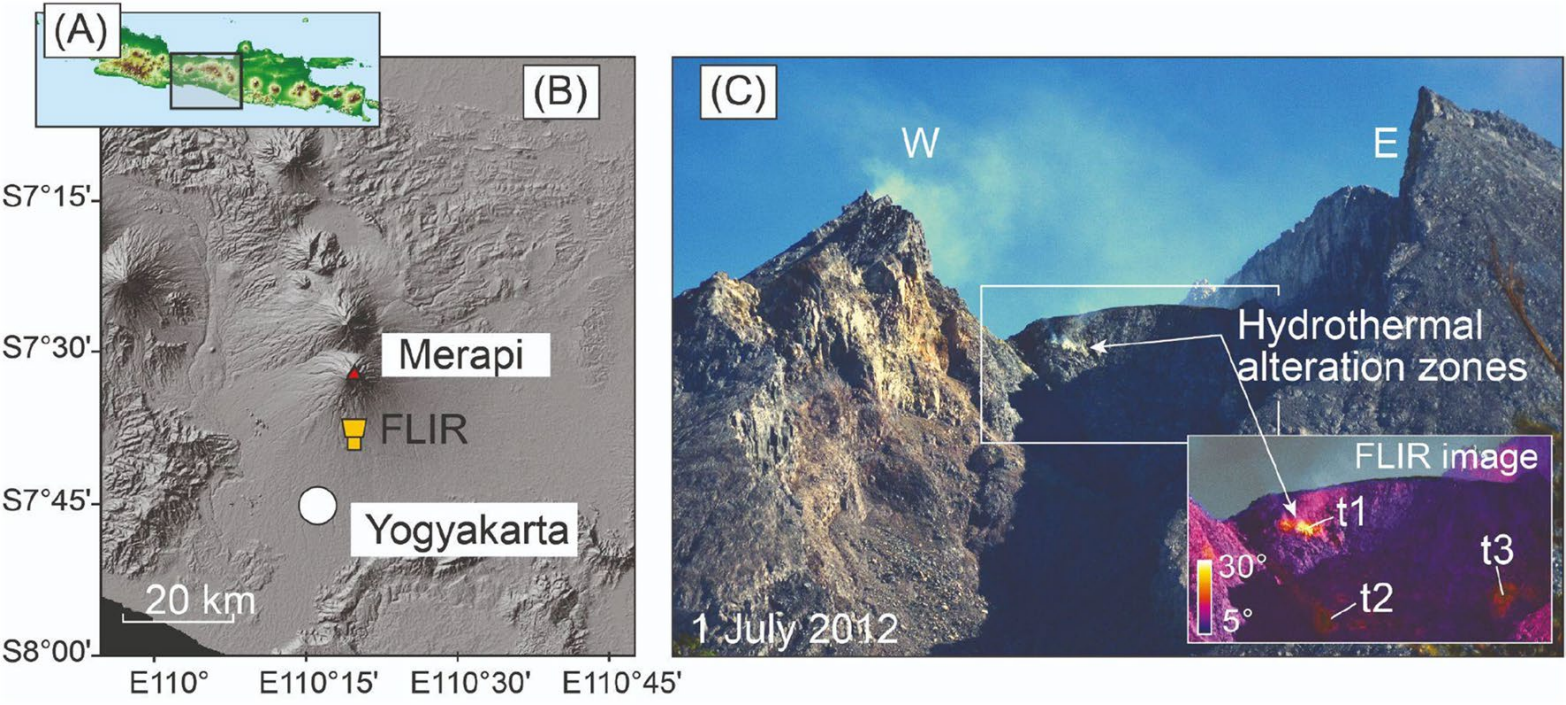
Merapi volcano. (**A**) Overview map of Java Island with Central Java marked by black box. (**B**) View of Merapi volcano from the South. White box marks enlarged area in subsequent image. (**C**) Merapi summit dome close up photograph showing fumarole activity and ongoing hydrothermal degassing including yellowish coloured alteration zones (1 July 2012). Inset in (**C**) shows a thermal infrared camera image (handheld camera type: FLIR P 660) that highlights apparent temperature highs located at the southern part of the lava dome (t1 and t2) prior to the 2012–2014 explosions. Mapviews were created using ArcMap (v10.5, https://desktop.arcgis.com/de/arcmap/), FLIR image created using the FLIR ThermaCAM Researcher Pro (vs2.10) software.

**Figure 2:**
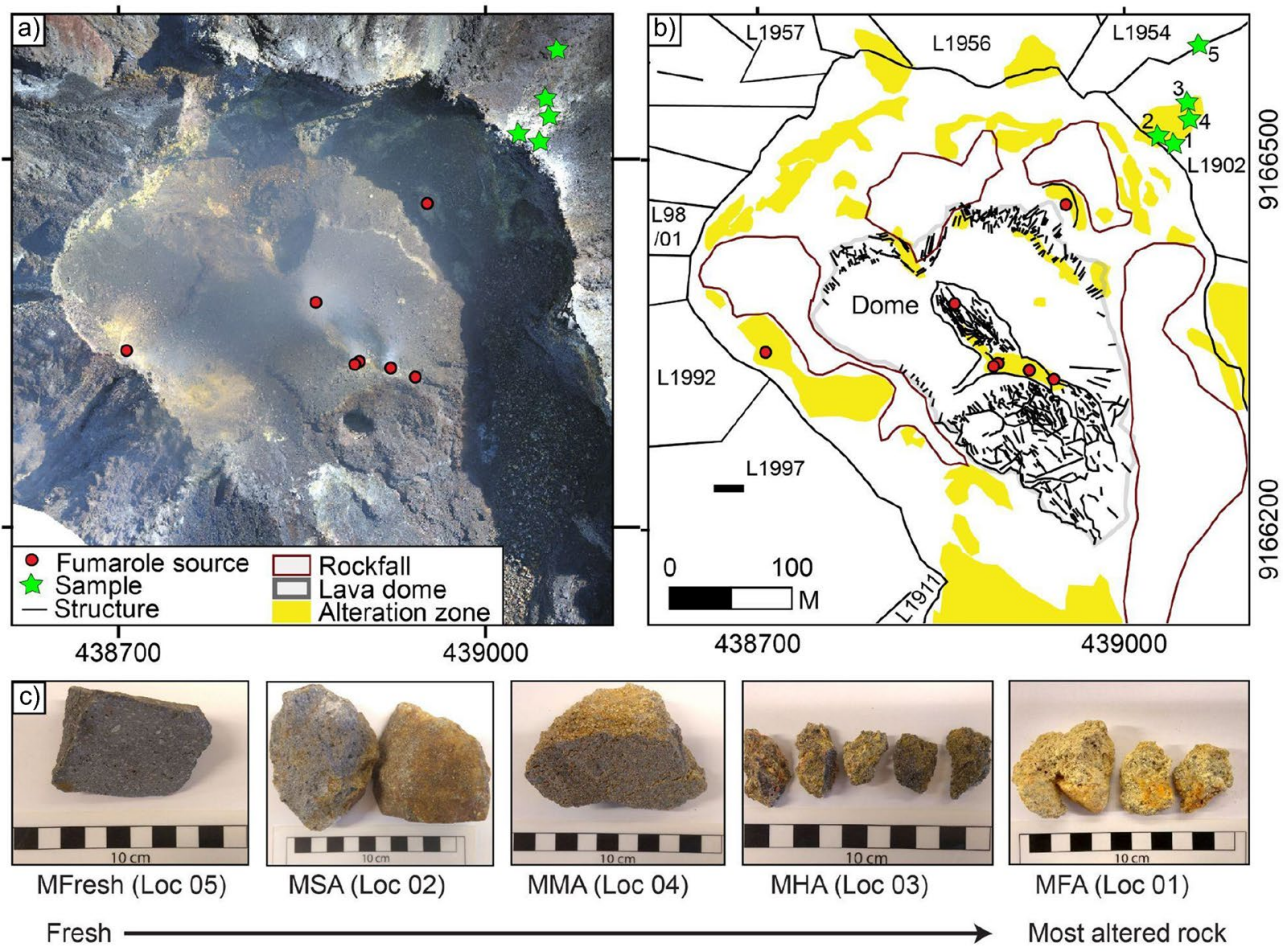
Hydrothermal alteration at Merapi summit in 2017. (**a**) Photomosaic of drone images acquired in 2017 used to map hydrothermal alteration at Merapi summit. No significant deformation was observed between 2015 and 2017, however, many rock falls were deposited at the western and the eastern crater floor area during this period. (**b**) Map of hydrothermal alteration, structures and active fumaroles at Merapi summit, and the sample location of the Merapi dome rocks that are used in this study (from the 1902 dome lava). (**c**) The Merapi dome rock samples show different degrees of alteration from fresh to intensely altered, as already identified by their colour changes. Mapviews were created using ArcMap (v10.5) (https://desktop.arcgis.com/de/arcmap/).

Merapi volcano (Java Island, Indonesia) is a major stratovolcano in Central Java and is part of the Sunda arc chain of active subduction zone volcanoes that includes the islands of Sumatra (West Sunda arc), Java, Bali and Flores (East Sunda arc)^20^. Subduction of the Indo-Australian plate beneath the Eurasian plate at a rate of up to 7 cm per year provides the driving force for the active volcanoes in the region. Merapi volcano is located in the Central part of the Sunda arc and erupts frequently due to dome collapses that at times generate devastating pyroclastic flows (i.e. “Merapi-type” eruptive style)^21^. Partial dome collapse and deep mechanical erosion associated with pyroclastic flows in 2010 left a deep open ravine that directed and channeled flow-hazards southwards towards the Yogyakarta urban area, forcing over 200,000 inhabitants to leave their homes^22^. Here we hypothesize that hydrothermal alteration in the Merapi dome plays a role in large-scale dome collapses and associated explosions and pyroclastic flow generation, because dome collapse deposits of 2006 and 2010, for instance, contain up to 46% hydrothermally altered fragments by volume^8,11,23,24^. The explosions that occurred from 2012 to 2014 and in 2018 were also located along weak NW–SE structures^17,25^ that showed high degrees of alteration, as seen in our thermal and high-resolution drone camera images (Figs. 1, 2). Our drone photogrammetry documents hydrothermally altered rockfall debris at the eastern and the western crater floor of Merapi during quiescence periods between late 2014 and 2017 (Fig. 2), highlighting that hydrothermal activity plays a crucial role in gravitational instability hazards at Merapi^26^. As hydrothermal activity and alteration progresses, instability is likely also evolving, so that existing structures become weaker, which may affect subsequent eruptive processes and phenomena. While gravitational instabilities operate on a timescale of weeks to months, recent work on altered Merapi dome samples postulated that the precipitation of secondary minerals can temporarily reduce dome permeability due to hydrothermal alteration (e.g. hours to days)^27^. This process could seal outgassing channels and promote a temporary build-up of gas pressure under the dome, which could be released in small-scale erratic explosions^27^. At the surface, such processes lead to localised fumarole activity that can be monitored using remote sensing data^25^. Therefore, processes observed at Merapi and the frequency of hydrothermal alteration recorded in clasts within its deposits suggest not only that hydrothermal activity plays a role in short-lived pressure build up at Merapi-type dome-building volcanoes, but also that it may be the crucial factor in causing progressive destabilization and subsequent large-scale gravitational dome collapses over somewhat longer time scales. Indeed, dome building at Merapi is frequently accompanied by hydrothermal fluid circulation that alters the originally fresh dome lavas at temperatures between ~ 50 and ~ 500 °C, as also observed at similar volcanoes elsewhere^6,25,28^. It has been shown that acidic hydrothermal alteration results in mineral replacement in volcanic rock which can reduce rock strength and internal friction^29–33^, which can, in turn, lead to a weakening of the volcano edifice internally or through the burying of altered and weakened surface dome rocks^34^. This phenomenon of buried hydrothermally altered structures of lower strength can therefore promote potentially catastrophic failure of summit domes and steep volcano flanks without direct visual indication at the dome surface. Moreover, large-scale collapses due to hydrothermal weak zones within the edifice may thus occur without significant precursory warning or unrest^4^, and may or may not be associated with simultaneous erratic explosive outbursts^27^.

In order to improve our understanding of the role and especially the location of hydrothermal alteration processes on volcano stability, we have investigated the Merapi dome through remote sensing, mineral and rock analyses, and compare the results to mechanical strength tests. To do so, we first identified hydrothermal alteration features and documented a secondary mineral accumulation at Merapi’s summit using repeated drone photogrammetry. We then collected a suite of hydrothermally altered dome rocks that preserve an increasing degree of alteration from fresh to intensely altered dome lava that marks the flanks of the active present-day dome area (Fig. 2 and Supplementary Table S1). These rock samples were assumed to represent the typical alteration sequence at the Merapi dome and were analyzed using wavelength dispersive X-ray spectroscopy (WDS) mapping, X-ray diffraction (XRD), and mass spectrometry for oxygen isotope ratios (reported as δ^18^O values). Based on visual inspection of the samples and the mineralogical and oxygen isotope data (see Fig. 2 and Supplementary Tables S1 and S2 for detailed rock and mineral descriptions), we categorized our dome rock samples as either fresh (MF), slightly altered (MSA), moderately altered (MMA), highly altered (MHA), or fully altered (MFA). Subsequently, porosity measurements and mechanical strength tests were performed on cylindrical core samples prepared from the dome sample blocks to analyze the relationship between hydrothermal alteration, porosity, and the mechanical strength of the dome rocks. We then combined the alteration and rock mechanical information with geoinformatics and our analysis of drone imagery to highlight the locations of hydrothermal alteration and progressive alteration at specific sites. We show that currently ongoing instability of the Merapi dome occurs along previously altered structures that were buried by renewed dome extrusions and act now as mechanical weaknesses within the Merapi dome complex.

## Results

### Remote sensing analysis of hydrothermal alteration

**Figure 3:**
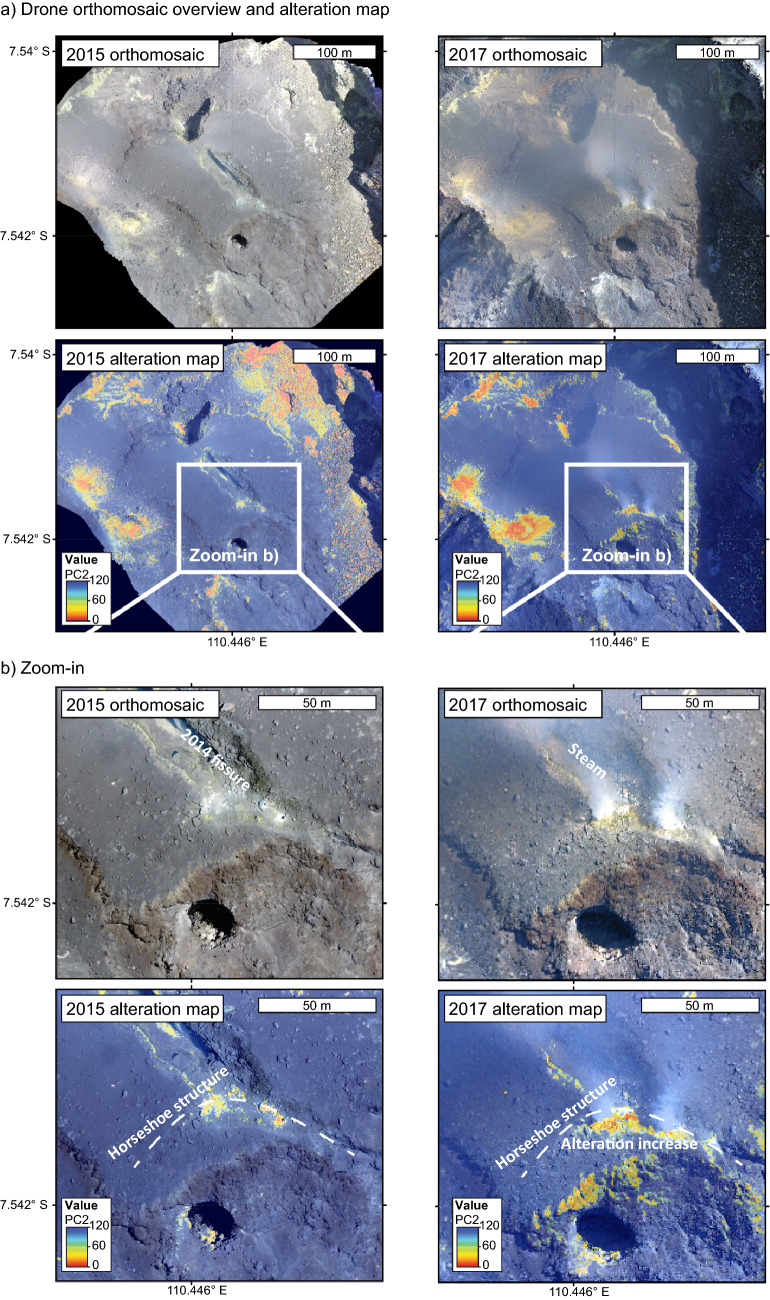
Temporal changes at Merapi summit dome. (**a**) Drone data was processed to generate high resolution orthomosaic representing the Merapi dome in map view (upper row) for 2015 (left) and for 2017 (right). The principle component analysis for PC2 is suggesting an increasing area of hydrothermal alteration. Shadowed regions are not further considered. White box indicates the area of the zoom-in. (**b**) Zoom-in of the orthomosaic and PCA maps, highlighting for the observed 2-year period, that the degree of steaming and alteration notably increases along the eastern segment of the horseshoe-shaped fracture, as well as at the southern flank of the lava dome. Note that this area has collapsed and is the site of frequent mass wasting events and the origin of pyroclastic density currents during the recent (post 2019) crisis of Merapi volcano. Orthomaps and PCA analysis were created using ArcMap (v10.5) (https://desktop.arcgis.com/de/arcmap/).

Quantifying the degree of hydrothermal alteration and identifying ore and mineral deposits through alteration mapping has been a main motivation for employing imaging remote sensing techniques at active volcanoes for several decades^35,36^. The monitoring of geothermal activity and associated mineral mapping has been widely applied, in particular multispectral and hyperspectral imagery has improved the visualization of hydrothermally altered regions^35,36^. While the strength are the spectral cameras, the main limitation of these techniques, however, is their resolution. The pixel-diameter of most modern spectral satellite imagery is exceeding 10 m scale, which does not provide a resolution appropriate for monitoring the evolution of alteration at volcanic domes. As a result of this shortcoming, recent studies have successfully utilized drone-based optical photogrammetry data at dome building volcanoes such as Merapi^25,37^. Drone imagery has also been used for image band combination, Structure-from-Motion (SfM), and image classification^38^. While many hydrothermal alteration effects are visible in the drone photos, the Principal Component Analysis (PCA) is an additional efficient statistical tool that can reveal information that is not accessible to the naked eye^39–41^. In a first step we use the drone image SfM approach for the identification of the geomorphology and dimensions of the evolving Merapi lava dome, revealing two main domes, a younger one covering an older one, both located inside the Merapi crater open to the South. In a second step we use PCA to detect and map hydrothermally altered features. The PCA approach allows to visualize even slight color variations through reorganization of the initial camera data along the perpendicular axes of their highest variance/covariance. For instance, the dimensionality reduction from 3 optical channels to single principal components, PC1, PC2 or PC3, allows us to separate and identify areas associated with brightness estimates (PC1) or alteration-typical greenish-yellowish colorization (PC2). Using this approach, we can indirectly identify characteristic pixel colorization through the PC2 that is likely associated with sulfuric deposition and acid sulfate alteration^41^. We carefully compared regions located outside the area of interest (the lava dome) and found that the number of pixels detected in PC2 in many parts was constant in 2015 and 2017, whereas a strong increase of the number of pixels from 800 m^2^ to over 2200 m^2^ was recorded along the eastern segment of a horseshoe -shaped fracture system and on the southern sector of the active lava dome (Figs. 2, 3). This finding is qualitative, however, especially since optical effects by hydro-meteorological conditions are commonly challenging for photogrammetric methods, but our drone analysis provides nevertheless a first-order estimate on the increase of sulfuric deposition and acid sulfate alteration. The area of the southern sector was covered by renewed dome lava extrusion in 2018, but subsequently collapsed and is now the site of frequent mass wasting events and the origin of pyroclastic density currents during the recent (post 2019) crisis of Merapi volcano^10,26,42^ (see also below).

### Changes to mineral assemblage

**Figure 4:**
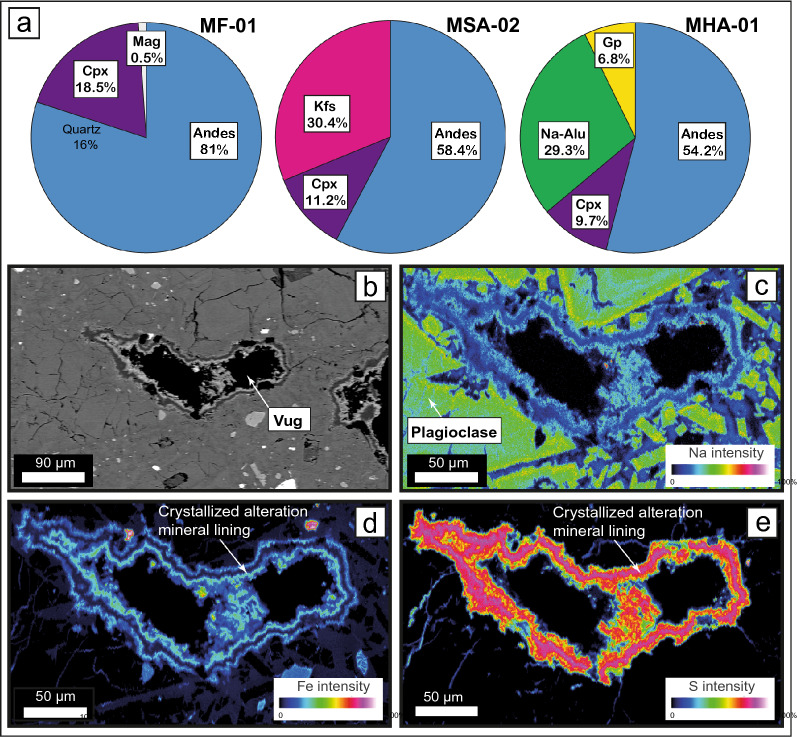
Mineralogy of dome lava samples. (**a**) Pie charts of mineral contents determined by XRD from fresh dome lava (left), moderately altered dome lava (centre) and strongly altered dome lava (right) based on data in Supplementary Table S2. The amount of andesine feldspar (mid- Ca plagioclase) is steadily decreasing while the proportion of alteration minerals (e.g. natro-alunite, gypsum) is seen to increase, documenting progressive replacement of the original rock mass with an acidic sulphurous alteration mineral assemblage. (**b**) BSE image of a lined vug in the strongly altered dome rock sample. (**c**–**e**) Chemical element maps (Na, Fe, and S respectively) showing Fe and S enrichment in the secondary minerals that are lining the vesicle, but low Na content relative to primary minerals. The combined mineralogical evidence from XRD and elemental mapping implies that hydrothermal alteration at Merapi progressively replaces strong silicate minerals (e.g. plagioclase) with sulfate mineralization and additional precipitations in fractures and vesicle spaces. While this will progressively reduce porosity of the altered rock, we show that the combined mineralogical changes will cause an overall decrease in the mechanical strength of the dome rock (see Fig. 5).

We sampled a series of variably altered dome lava specimens from the crater margin, which were extruded as part of the AD 1902 lava dome (Fig. 2). We employed these samples to better understand the influence of progressive hydrothermal alteration on lava composition and properties (see below). In respect to compositional changes, our XRD data (Supplementary Table S2) indicate that progressive hydrothermal alteration first reduces the amount of primary feldspar and pyroxene in the dome rock, and then promotes the growth of secondary alteration minerals (natroalunite, gypsum, jarosite, and hematite). The fresh Merapi dome lava sample (MF), which is dark grey in colour, has a dry bulk density of ~ 2580 kg/m^3^. Sample MF has an abundance of plagioclase phenocrysts with pyroxene and minor amphibole and magnetite crystals (see Fig. 4 and Supplementary Tables S1 and S2), contains 80% (reported as vol. %) original magmatic andesine (medium Ca plagioclase feldspar), 19% clinopyroxene (cpx), and 0.5% magnetite (mt). Several other minerals were observed in small proportions in thin sections, but were not recorded in XRD patterns (e.g. amphibole). The slightly altered dome lava sample (MSA) has a dry bulk density of ~ 2500 kg/m^3^ and contains less andesine feldspar and clinopyroxene (58 and 13%, respectively), but notably contains about 30% potassium (K-) feldspar, indicating the first stages of conversion of the primary plagioclase mineralogy from the fresh dome lava sample. The moderately and highly altered dome lava samples (MMA–MHA) show more intense changes in mineralogy with the moderately altered dome lava sample (MMA) containing 76% andesine, 11% clinopyroxene, and 11% sulfate alteration minerals (natroalunite) and ~ 1% hematite, while the highly altered dome lava sample (MHA) contains 51% andesine, 9% clinopyroxene, and 41% sulfur-bearing alteration minerals (31% natroalunite and 10% gypsum). The dry bulk densities of MMA and MHA are ~ 2190 and ~ 2130 kg/m^3^, respectively. Finally, the fully altered dome lava sample (MFA) contains the highest amount of alteration derived (63%) high-Ca (anorthite) feldspar, 10% clinopyroxene, and over 20% sulfur-bearing alteration minerals (11% natroalunite and 12% jarosite) and has a dry bulk density of ~ 2100 kg/m^3^. These mineralogical changes together with micron-scale elemental mapping of partly-filled cavities in the altered Merapi lava dome sample (Fig. 4) underscores that hydrothermal alteration leads to volumetric conversion of the original magmatic mineral assemblage (mainly plagioclase and clinopyroxene with minor magnetite), to progressively more pronounced alteration assemblages (potassium feldspar, anorthite plagioclase hematite, natroalunite, jarosite, and gypsum)^27,43^ as well as the infilling of cavities and fractures with layers of sulfur (S) and iron (Fe)-rich alteration products (Fig. 4). Indeed, chemical element maps for Na, Fe, and S show Fe and S enrichment in the secondary minerals that are lining vesicles, but low Na content relative to primary minerals.

### Lava dome oxygen isotope (δ^18^O) values

To quantify the degree of hydrothermal alteration in our samples, we analyzed their whole-rock oxygen isotope ratios (reported in standard delta notation, δ^18^O)^44^ and combined these with of the δ^18^O value for a condensed fumarole H_2_O fluid sample at Merapi summit (δ^18^O = − 14.75 ‰; Supplementary Table S2)^45^. Using these data, we calculated relative water–rock ratios and equilibrium alteration temperature for each sample. The water–rock ratios (*W*/*R*) were calculated using the equation:

1 $$\frac{W}{R}=\frac{{\delta }_{Rock}^{f}-{\delta }_{Rock}^{i}}{{\delta }_{{H}_{2}O}^{i}-\left({\delta }_{Rock}^{f}-\Delta \right)}$$

where ${\delta }_{Rock}^{f}$ is the δ^18^O of the altered rock, ${\delta }_{Rock}^{i}$ is the δ^18^O of the unaltered rock, ${\delta }_{{H}_{2}O}^{i}$ is the measured of δ^18^O of the fumarole fluid at the summit of Merapi, and $\Delta$ =${\delta }_{Rock}^{f}$ − ${\delta }_{{H}_{2}O}^{f}$ (see Taylor, 1977)^46^. The temperature of hydrothermal alteration was estimated using equilibrium fractionation factors for sulfate minerals^47^:

2 $${1000ln\alpha }_{i-j}=\frac{A \times {10}^{6}}{{T}^{2}}+\frac{B \times {10}^{6}}{T}+C,$$

where α_*i-j*_ is oxygen the isotope fractionation of two substances, *A*, *B*, and *C* are constants following the study of Seal et al.^47^, and *T* is the temperature in *K*.

Hydrothermal alteration increases the whole rock δ^18^O value from + 7.2 to + 12.7 ‰ (Supplementary Table S2). The low-porosity fresh lava dome sample (MF) yields δ^18^O values from + 7.2 to + 7.6 ‰ (± 0.15%), similar to magmatic δ^18^O values for Merapi (+ 5.7 to + 8.3)^44,48^ and negligible water–rock interaction (water–rock ratio = 0). The δ^18^O values of the slightly altered lava dome samples (MSA) fall around + 8.3 ‰ and thus correspond to a water–rock ratio of 0.2. The δ^18^O values then increase progressively from + 10.7 to + 12.7 ‰ in the moderately (MMA), highly (MHA), and fully (MFA) altered lava dome samples, and the water–rock ratios of these samples were calculated to range from 0.7 to 1, which is associated with a temperature range from 147 to 238 °C (see Supplementary Fig. S1). This temperature range is consistent with our thermal image dataset recorded in 2014^25^, which gave temperatures > 140 °C.

### Changes to rock strength

**Figure 5:**
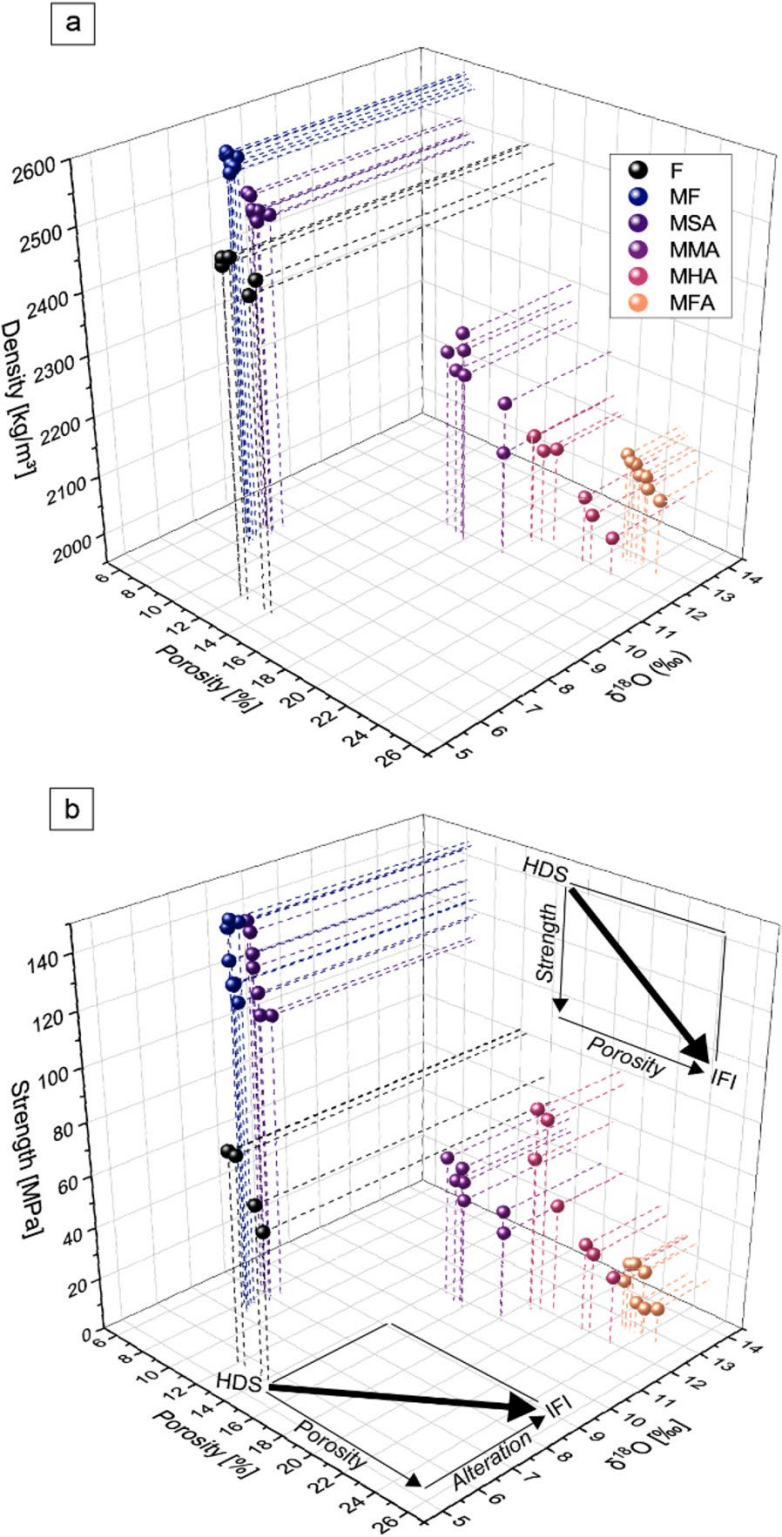
The influence of porosity and hydrothermal alteration on rock strength. (**a**) Density as a function of porosity (%) and oxygen isotope compositions (δ^18^O) as a proxy for degree of alteration for the variably-altered rocks collected from the Merapi dome. The unaltered but porous samples from the 2006 eruption are also shown (black symbols; F = fresh). Dotted lines are aids to help visual orientation. (**b**) Uniaxial compressive strength (MPa) as a function of porosity (%) and oxygen isotope compositions (δ^18^O) as a proxy for degree of alteration for the variably-altered rocks collected from the Merapi dome. Rock strength decreases as a function of degree of alteration and porosity (see also Supplementary Fig. S1) implying that edifice stability is controlled by a combination of porosity and hydrothermal alteration (see inset). *HDS* high dome stability, *IFI* increased flank instability.

Testing the lava dome samples for uniaxial compressive strength (UCS) shows that mechanical strength in our sample suite is lessened as a function of increasing porosity and degree of hydrothermal alteration (Fig. 5; Supplementary Table S3). The porosity of the studied dome samples varies from 8 to 24%, and their strength varies from 132 to 19 MPa (Fig. 5a). Furthermore, we find that the fresh lava dome sample (MF) is the strongest with an average UCS of 132 MPa, while the slightly altered (MSA), the moderately altered (MMA), and the highly altered (MHA) dome samples have an average UCS of 125, 46, and 49 MPa, respectively. Finally, the fully altered dome rock (MFA) is the weakest and has an average UCS of 11 MPa. We performed additional control experiments on a fresh block sample from Merapi’s 2006 eruption that preserved a higher porosity (13%) than the fresh dome sample (MF) in our lava dome sample suite (porosity of 8%; UCS of 73 MPa). Therefore, we observe that either increasing hydrothermal alteration, increasing porosity, or both can subsequently reduce rock strength (Fig. 5) and can, in conjunction, decrease the strength of dome rock from Merapi by a factor of about 10.

Although the observed alteration has been shown to result in local decreases in porosity and permeability within these dome rocks^27^, such alteration need not necessarily result in an overall increase in strength (as porosity-strength relationships for volcanic rocks would suggest; see Ref.^49^). Indeed, our results show that the observed alteration not only causes a decrease in permeability within the blocks^27^, but also a decrease in strength relative to an unaltered block.

## Discussion

Our multifaceted analytical approach reveals a strong relationship between the formation of hydrothermal alteration minerals, the porosity of dome lava, and the lowering of mechanical strength, and implies, in combination with our remote sensing information, that hydrothermal processes have a strong influence on dome instability and failure sites.

### Acid-sulfate hydrothermal mineral alteration

Interaction between acidic hydrothermal fluids and the dome rock at Merapi produces an alteration mineral assemblage that comprises Ca-rich (anorthite) plagioclase, hematite and abundant sulfate mineral phases such as natroalunite, jarosite, and gypsum (Supplementary Table S2; see also Ref.^27^). This sulfate-rich alteration mineral assemblage develops at the expense of the original high-temperature mineral assemblage in the fresh magmatic rocks (medium Ca andesine plagioclase, pyroxene, magnetite, and minor amphibole) and is typical for dome alteration in andesitic stratovolcanoes (e.g.^27,43^). Natroalunite forms in a sulfuric-acid environment due to the disproportionation of SO_2_ in a condensing magmatic vapor below temperatures of ~ 350 °C, following the reaction 4 SO_2_ + 4 H_2_O = 3 H_2_SO_4_ + H_2_S^50^. The presence of natroalunite at up to 30% thus confirms the localized influence of high-temperature SO_2_–rich acidic fluids at the Merapi dome. At lower temperatures, and at short distance to high temperature fluid venting sites, alunite may be associated with the oxidation of iron to form hematite^51^ as is indeed found in our moderately altered rock sample. The magmatic SO_2_ gas that flows upwards interacts with meteoric fluids^52^ causing intensive oxidation of H_2_S. This phenomenon reduces the amount of alunite and, as a consequence, jarosite forms under fumarolic conditions, which is known to occur at temperatures as low as ~ 120–250 °C and a pH of 1–4 (Ref.^53^). Gypsum formed in the most intensely affected samples, which is considered to result from dissolution and/or re-precipitation of soluble sulfate around the central portions of the fumarole venting areas at temperatures below c. 60 °C (Ref.^43^). We thus see the influence of two sources of fluids at Merapi, magmatic and meteoric, due to the formation of natroalunite-hematite-gypsum-jarosite. This dual magmatic-meteoric fluid regime at Merapi is probably also reflected by low resistivity areas at depths down to 200 m, which is meteorically dominated, and again at ~ 1800 m below the summit, which is magmatic-hydrothermally dominated^54^.

The combined mineralogical evidence from XRD and elemental mapping thus implies that hydrothermal alteration at Merapi progressively replaces strong silicate minerals (e.g. plagioclase) with sulfate mineralization and produces additional precipitation of alteration minerals in fractures and vesicle spaces (Fig. 4). While this will progressively reduce porosity of the altered rock, we show that the combined mineralogical changes will cause an overall decrease in the mechanical strength of the dome rock (see Fig. 5).

### Oxygen-isotope exchange

The physiochemical dissolution and precipitation reactions during acid hydrothermal alteration cause an exchange of the light oxygen isotope for the heavy oxygen isotope, and thus whole-rock δ^18^O values gradually increase during progressive alteration (at determined temperatures of 147–283 °C). The observed increase of δ^18^O values in our samples correlates with an increase of the abundance of alteration minerals and the δ^18^O value in our dome samples therefore reflects an increase in water–rock ratio and the temperature of hydrothermal fluids that altered the dome lava samples we analyzed. Specifically, fresh Merapi bulk rock has δ^18^O values of + 6.4 to + 8.3‰ (Refs.^44,48^), which is typical for mantle-derived magma that has undergone various degrees of crustal contamination in a continental arc setting (the Sunda arc is mixed oceanic-continental, with relatively thick crust underlying Merapi^20,55^). The moderate to fully altered dome rock samples yield much higher whole-rock δ^18^O values of + 10.8 to + 12.8 ‰ and thus reflect an increase in water–rock ratios (0.7–1). This increase in δ^18^O values and water–rock ratios in our suite of rock samples implies alteration temperatures of up to ~ 280 °C (Ref.^56^), which is consistent with a thermal investigation in 2014 that suggested hydrothermal alteration surface temperatures at the Merapi lava dome around or in excess of 135 °C (Ref.^25^), and determines the usefulness of remote temperature determination in recording the location of alteration in the dome area. Moreover, the changes in oxygen isotope ratios are correlated with changes in density (Supplementary Table S3), implying that mineral replacement of the original denser magmatic minerals by less dense secondary sulfate minerals is an important factor. The calculated results imply that approximately half of the oxygen in the most hydrothermally overprinted rock was replaced by oxygen from hydrothermal fluids at a lower temperature. This was accompanied by the formation of the sulfate-dominated alteration assemblage within the investigated sample suite (Supplementary Table S2; Fig. 4) and underlines the internal changes that take place during acid hydrothermal alteration, whereby large portions of the original rock mass are effectively replaced by components derived from the percolating acidic fluids that pass through the permeable lava dome.

### Mechanical weakening promotes gravitational failure at “Merapi-type” dome volcanoes

Hydrothermal alteration is often considered to lower mechanical rock strength due to the breakdown of the original mineral framework and the formation of mechanically weaker alteration minerals^30–33,57^. Our results indicate that the strength of the variably altered Merapi dome samples correlates with porosity and hydrothermal alteration intensity (Fig. 5). Indeed, increasing porosity^58,59^ and more intense hydrothermal mineral alteration^32,57,60,61^ have been previously shown to lower the strength of volcanic rock (see also recent review Ref.^49^). However, based on our new data (Figs. 4, 5), it is difficult to directly assess the influence of hydrothermal alteration on rock strength. This is because the relationship between δ^18^O, a proxy for alteration, and strength can also be explained by the fact that the more porous rocks are more altered as a result of their higher effective fluid-rock ratio. In other words, it is not possible with the current dataset to confirm whether the hydrothermal alteration increased the porosity of these materials, resulting in a decrease in strength, or whether the more porous rocks are simply more altered as it permitted higher rates of fluid flow. To better understand to what extent porosity or hydrothermal mineral replacement is controlling the observed mechanical behaviour, we performed additional experiments on a fresh block sample from Merapi’s 2006 eruption that preserved a higher porosity (13%) than the fresh dome sample (MF) in our sample lava dome sample suite (porosity of 8%). The average UCS of the unaltered 2006 higher-porosity sample is 73 MPa, almost a factor of two higher than the similarly porous moderately altered dome lava sample (16% porosity at 46 MPa, Fig. 5a), underpinning the impact of alteration on rock strength. We conclude, therefore, that alteration reduced the strength of our Merapi dome lava samples and that it is likely that the more porous samples have experienced larger decreases to their strength as a result of their higher degree of alteration. In detail, for a given porosity, a reduction in UCS is seen due to the difference in strength between primary minerals and alteration minerals. The more than ten-fold reduction in rock strength measured in our dome lava samples thus represents the compound effects of porosity increase and acid-sulfate alteration. As a result, our new strength data show that, although pore- and microcrack-filling alteration can reduce permeability and potentially promote erratic explosive behaviour^27^*,* the replacement of strong primary minerals with weak alteration minerals ultimately results in a decrease in strength. This conclusion is consistent with recent work that showed that microcrack-filling clays can reduce the strength of andesite by reducing the coefficient of internal friction^29^. We conclude, therefore, that the combined effect of porosity and hydrothermal alteration exerts a fundamental control on dome stability at Merapi and likely other active Merapi-type volcanoes. Indeed, if alteration of the lava dome at Merapi can reduce both permeability^27^ and strength (this study), both of which can destabilize the dome, then understanding the extent and evolution of alteration emerges as an essential component of volcanic hazard assessment.

### Implications for current and future instability at the Merapi dome complex

**Figure 6:**
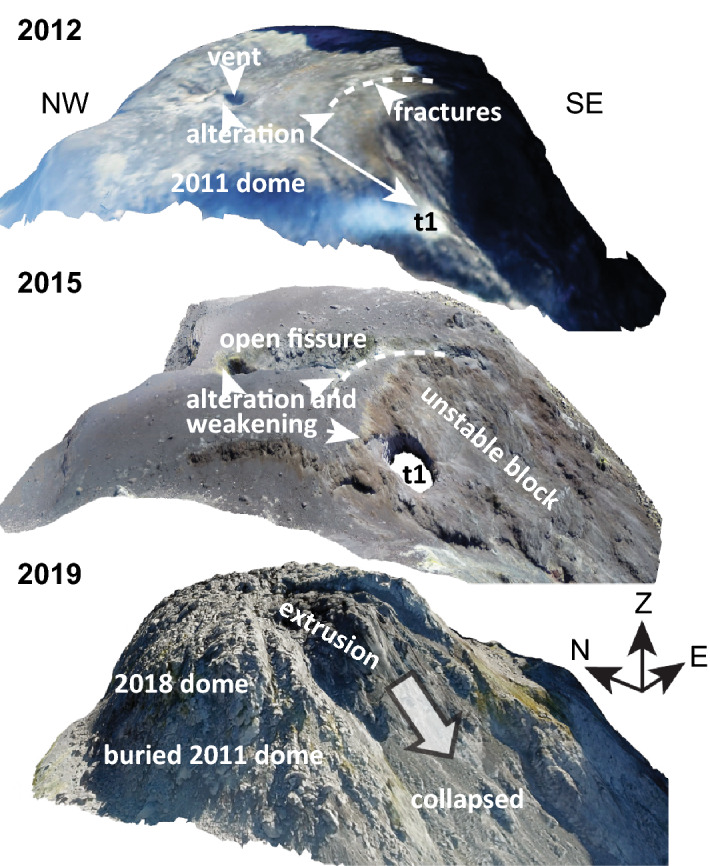
Oblique view of the 3-D rendered model of the Merapi lava dome, imaged by drone cameras in 2012, 2015 and 2019. The thermal anomaly spot (t1) and the horseshoe-shaped fracture in 2012 has given rise to the site of an explosion crater in 2015. A horseshoe-shaped open fissure formed in 2014 and is visible in the image from 2015. The 2019 data show the new lava dome, mantling and burying the earlier dome structures. This new lava dome erupted in 2018 and began to collapse in 2019 along the fracture system that developed in 2014. The total volume of the 2018/19 dome is assumed to have been twice as large as shown by the 2019 image due to frequent material losses^10^. Our data imply that the buried 2014 hydrothermally altered fracture system presently exerts a fundamental control on dome stability and associated rock falls and pyroclastic density currents at Merapi. t1 is a local crater that evolved at the high temperature and alteration area seen in 2012 already (cf. Fig. 1). Oblique views were created in Agisoft Metashape (v1.7) (www.agisoft.com).

Our findings indicate that alteration related weaknesses play a role in the instabilities of the southern sector of the Merapi lava dome^25^. Direct on the ground monitoring of the progressive changes caused by alteration is challenging, but by considering the visual emergence and intensification of sulfuric depositions in the southern sector of the dome as early as 2012, we can now provide further constraints on the spatial progression of the hydrothermal activity and the influence this activity may have on current dome instability. This same sector was shown to have been progressively hydrothermally altered prior to 2017 (Fig. 3) and then partially collapsed producing a hazardous pyroclastic density current in 2019 (Refs.^10,26,42^). Moreover, this area is also the site of frequent mass wasting events, and thus the origin of pyroclastic density currents, during the most recent (post 2019) crisis at Merapi volcano. In detail, the total eruption volume of the 2018/19 dome is assumed to have been twice as large as shown by the 2019 images due to frequent material losses^10^ and our data imply that the buried 2014 hydrothermally altered fracture system still exerts a fundamental control on dome stability and associated rock falls and pyroclastic density at Merapi at present. This means the buried, and thus hidden, hydrothermally weakened and fractured rocks were most probably the main reason for localizing the recent and ongoing partial dome collapses at this specific site (Figs. 5, 6). These realizations on pre-2017 developments in combination with the recent 2018–2019 lava dome extrusion and collapse events at Merapi, allow us now to identify key locations under the active Merapi dome that are likely to act as failure sites due to alteration effects in the near future. In addition, our findings now provide a conceptual means to identifying similar mechanically weak zones through consistent observations at Merapi and similar volcanoes in other parts of the globe.

More specifically, to explain the gravitational collapse of the southern block of the Merapi dome during the late 2018 and 2019 dome growth episode^10^, we consider a number of contributing factors. Importantly, the newly collapsed area was delineated by the weakened crescent-shaped structure that formed already in 2014 (Figs. 3, 6), but was buried by the 2018/2019 lava dome extrusion. During this latest dome growth episode in 2018/2019, pore pressure and shear stresses on this delineating structure would have likely increased^10,62^, while newly extruded dome material would have added to the load on this previously formed fracture and hydrothermally weakened dome sector. The weakened dome sector then collapsed in early 2019 as indicated by our drone image (Fig. 6), an event that was followed by series of gravitational collapses of, and associated rockfalls from, the growing new dome between 2019 and 2020, which also produced small-scale pyroclastic flows on the south eastern flank of Merapi^26,42^.

Drone based optical photogrammetry data containing pixel-wise information in RGB space can therefore not only provide the topographic dataset showing a nested dome structure (Fig. 6), but also provide a qualitative indication for both alteration and sulfuric deposition effects^41^. Repeat drone data at Merapi volcano from 2014 to 2017 suggests an intensification of alteration processes in the sulfuric-yellowish areas (Fig. 3). Applying PCA to these data will eventually allow us to quantify the intensity of alteration in such alteration zones^41^, as the progressively increasing degree of dome rock alteration was clearly recognizable. These rocks were eventually covered by new dome lava effusions in December 2018 and thereafter.

Our work thus highlights that porous and hydrothermally weakened rocks on previous collapse surfaces can reduce the stability of growing volcanic edifices by introducing structural instabilities, and that mechanically weak hydrothermally altered structures hidden by younger deposits are important for controlling later sites and scales of instability at active dome volcanoes. Although these weak zones are challenging to locate beneath active and growing volcanoes, continual remote sensing and drone-based methods have the potential to closely observe their temporal evolution and record sites and intensity of zones of hydrothermal activity. For instance, when edifices are accessible, geophysical methods such as electrical tomography can identify and map out alteration zones^15,54,63,64^, but many active domes are inaccessible and alteration zones must be monitored using airborne methods such as photogrammetry^25^ and hyperspectral imaging^65^. We predict that future studies will be able to realize the monitoring of hydrothermal changes via drone surveillance on larger datasets, either from more frequent drone surveys or from installed fixed position monitoring cameras^10^. This creates enormous potential for our principal approach to become a vital monitoring tool in future eruptive episodes at Merapi and at similar dome-forming volcanoes worldwide.

The important realization for the Merapi dome from this study is that structural weaknesses can become integrated into a volcanic edifice by burial and, if not continuously monitored, can become a hidden weakness within a lava dome or edifice. The lessons learned at the Merapi during the run up of the 2019 dome collapses are likely relevant for similar andesite dome volcanoes elsewhere and long-term monitoring of structural features and associated hydrothermal alteration will help to identify ‘built in’ (and usually hidden) weaknesses in case of recurring dome extrusions. This approach will help us to better assess dynamically evolving lava domes that will otherwise present extremely challenging and potentially unpredictable instability phenomena at active lava dome volcanoes. We therefore put forward that the extent and location of buried alteration zones should be documented and incorporated into predictive models designed to assess volcano instability and its associated consequences.

## Materials and methods

### Drone-based mapping

We conducted repeated drone photogrammetry mapping at Merapi summit in 2015, 2017, and 2019 by using DJI Phantom 4 and Mavic pro. The drone flew ~ 500 m above the Merapi lava dome and captured in total over 1000 images from which we selected 359, 408, and 175 aerial images during the drone field campaigns in 2015, 2017, and 2019, respectively^25,26,37^. The drone datasets were used to reconstruct high-resolution Digital Elevation Model (DEM) and orthomosaic by applying the Structure-from-Motion (SfM) algorithm using the Agisoft Metashape workflow, yielding orthomosaics of pixel resolutions between 0.04 and 0.38 m. We then georeferenced the 2015, 2017, and 2019 datasets to the 2012 georeferenced 3D point cloud by using ground control points pair picking technique, yielding errors of ~ 1.0–1.5 m. The DEMs and orthomosaics provide realistic geometry and structures associated with hydrothermal alteration in 2015, 2017, and 2019 that can be used as the main parameter in the numerical modelling of dome instability.

For a principal component analysis (PCA) we work with the orthomosaics already described in earlier publications (e.g. Ref.^25^) and extended by the new overflights realized in 2019. The orthomosaics are referenced with respect to the 2012 dataset, the brightness was adjusted and the data constantly resampled to a mean pixel dimension of 0.5 m. We used the ArcGIS Geo-Information System (GIS) in the WGS84 projection and applied the PCA as implemented in the image classification toolbox. PCA is a tool used for dimensionality reduction, transforming the initial data (RGB) values onto the perpendicular vectors of its highest variance. With this approach we achieve a decorrelation of the initial RGB information and a variance representation, highlighting even slight variations of the surface colorization stored in the individual Principal Components (PC). PC1 and PC2 represent the highest variance of approximately 90% and 5–7%, respectively. By representation of the PC2 we follow earlier studies (e.g. Ref.^41^) to show areas of hydrothermal alteration and deposition, illustrated in Fig. 2.

The drone surveys and PCA provide a first order estimate of the area affected by hydrothermal alteration. For mapping alteration, the PCA technique is a widely used technique, and aims to extract specific spectral responses that are a consequence of hydrothermal alteration minerals^66^, PCA applied on optical data is to be interpreted with care, and results are not as robust as PCA applied to multispectral data^40^. Moreover, our data is notably affected by extrinsic changes. For instance, the PC1 may be considered for characterising sun illumination effects, so that photogrammetric data typically clusters along the main PC orientation from RGB (0 0 0) black to (255 255 255) white. Changes in sun illumination or shadowing would shift towards the white or black part, respectively. Consequently, sun illumination associated would affect PC1, whereas sulfuric deposition and acid sulfate alteration are likely to be detected in PC2 or PC3. As we demonstrated, the PC2 analysis from repeat surveys thus can allow for efficient monitoring and, through the consideration of the Eigenvectors/Eigenvalues of the principal components, can allow for first-order probing of the fumarole activity that is more robust than bare eye. Although the method has strong potential, further studies are needed for validation of the data and PCA approach.

### Sample preparation

The rock samples were cut at Uppsala University, Sweden, and slices were prepared for thin sectioning for mineral element mapping. An aliquot of each rock sample was crushed and milled using a jaw crusher and a hand-held agate pestle and mortar to produce sample powders that were subjected to X-ray diffraction (XRD) and oxygen isotope (δ^18^O_WR_) analyses (see below). Larger blocks of the samples were subsequently cored at the University of Strasbourg, France, to perform porosity, density, and uniaxial compressive strength (UCS) experiments. For these measurements, seven cylindrical samples (20 mm in diameter and nominally 40 mm in length) were cored from each of the sample blocks and then precision-ground so that their end-faces were flat and parallel. After coring and grinding, the samples were washed and then dried in a vacuum oven at 40 °C for at least 48 h prior to analysis.

### X-ray diffraction (XRD)

Between 1 and 3 mg of whole rock powder was used to determine the mineral composition of each sample through X-ray diffraction (XRD). The analysis was conducted using a PANalytical X’pert diffractometer equipped with an X’Celerator silicon-strip detector at the Department of Geoscience, Swedish Museum of Natural History, Stockholm. The instrument was operated at 45 kV and 40 mA using Ni-filtered Cu-Kα radiation (λ = 1.5406 Å). Samples were run between 5° and 70° (2θ) for 20 min in step sizes of 0.017° in continuous scanning mode while rotating the sample. Data were collected with "divergent slit mode" and converted to "fixed slit mode" for Rietveld refinement. The collected data show several peaks of X-ray diffraction intensity which represents the characteristic of crystalline minerals, which were then interpreted using the Rietveld refinement method in the High Score Plus 3.03e software. The XRD analytical procedure was performed twice for each sample to ensure optimal quality control.

### Element mapping

Chemical element maps of the altered rock samples were acquired on carbon coated thin sections using a Jeol JXA8530F Hyperprobe Field Emission Gun Electron Probe Microanalyser (FEG-EPMA) equipped with five spectrometers at the Department of Earth Sciences, Uppsala University, Sweden. Major mineral phases were first identified in altered samples based on EDS measurements. Concentrations of potassium (K), sodium (Na), sulfur (S), and iron (Fe) in major alteration minerals were mapped by WDS analyses of altered rock samples. The resulting chemical element maps of the altered rocks provide detailed distribution and intensity of the selected elements in the altered area.

### Oxygen isotope ratios of whole rock (δ^18^O) and water–rock ratio calculations

Powdered whole-rock sample aliquots (n = 15) were analyzed for oxygen isotope ratios using a Thermo DeltaXP mass spectrometer at the University of Cape Town, South Africa. Approximately 10–20 mg of material was dried under vacuum in nickel reaction vessels, then reacted with 30 kPa of CIF_3_ for 2–6 h to extract oxygen^67^. The extracted oxygen was converted to CO_2_ by passing it over to a high temperature platinized carbon rod. For full analytical details see Vennemann and Smith^68^ and Harris and Vogeli^69^. Unknowns were run with duplicates of the internal quartz standard (MQ) which was used to calibrate the raw data to the SMOW (Standard Mean Ocean Water) scale, using a δ^18^O value of 10.1 for MQ (calibrated against NBS-28). The results are reported in standard δ-notation, where δ = (R_sample_/R_standard_ – 1) × 1000, R_sample_ is ^18^O/^16^O in the sample and R_standard_ is ^18^O/^16^O relative to Standard Mean Ocean Water (SMOW)^70^. The analytical error is estimated as ± 0.2 per mil (2 sigma), based on long-term repeated analysis of MQ.

### Physical property measurements

We measured the porosity, density, and uniaxial compressive strength of each of the prepared 20 mm-diameter drill core samples (n = 35) to investigate how the physical changes of the rocks relate to their degree of hydrothermal alteration. The connected porosity of each sample was measured by using the bulk volume (determined using the sample dimensions) and the connected (skeletal) volume given by helium pycnometry (Micromeritics AccuPyc II 1340). The dry bulk density of each sample was obtained by simply dividing the dry mass and bulk volume of each sample. The unconfined compressive strength (UCS; σ_1_ > σ_2_ = σ_3_) was then measured using a uniaxial load frame. Oven-dry samples were deformed at an axial strain rate of 10^–5^ s^–1^ until macroscopic failure occurred. A lubricating wax was placed on the end-faces of the samples to avoid problems with friction between the sample and the pistons. During deformation, axial displacement and axial load were measured using a linear variable differential transducer and a load cell, respectively. These values were converted to axial strain and axial stress using the sample dimensions. For the full procedure, see ref^71^.

## Supplementary Information

Supplementary Information.
