# Supplementary material for: Hidden mechanical weaknesses within lava domes provided by buried high-porosity hydrothermal alteration zones
Source: Sci Rep. 2022 Feb 25;12:3202. doi: 10.1038/s41598-022-06765-9 (PMC8881499; doi:10.1038/s41598-022-06765-9)
Supplement: Supplementary file 1 — Supplementary Information. [file 41598_2022_6765_MOESM1_ESM.docx]

**Supplementary Material Darmawan et al.**


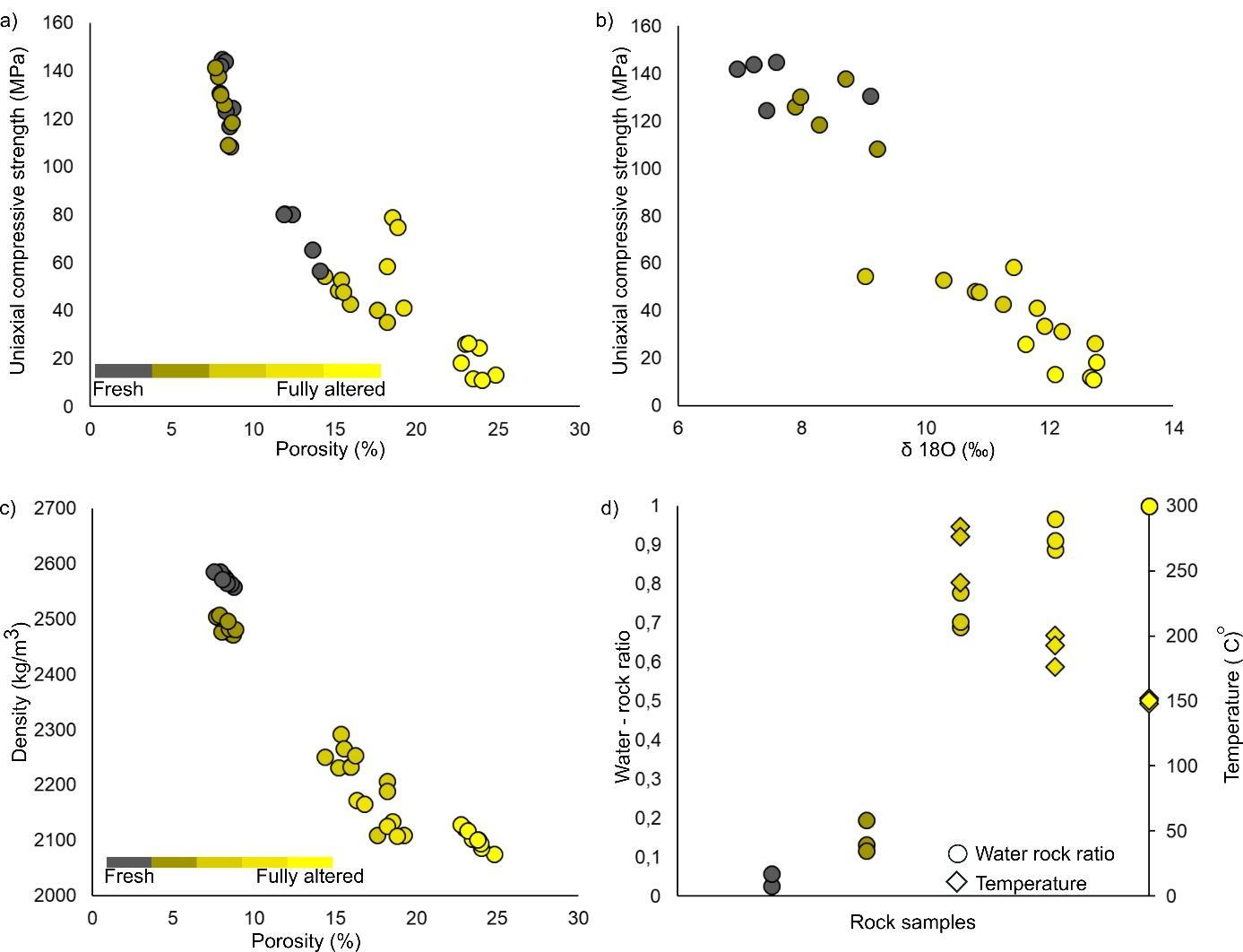


**Supplementary Figure 1. The influence of porosity and hydrothermal alteration on rock strength. (a)** Uniaxial compressive strength as a function of porosity for variably-altered rocks collected from the Merapi dome. The colour of the symbols indicates their alteration intensity, from fresh to fully altered. **(b)** Uniaxial compressive strength as a function of δ^18^O value for variably-altered rocks collected from the Merapi dome. Rock strength is controlled by a combination of porosity and hydrothermal alteration (see text for details). **(c, d)** Correlation between density changes, water – rock ratio, and temperature gradient due to hydrothermal alteration at Merapi. **(c)** Porosity can reduce the rock’s density from 2586 to 1972 kg/m^3^. **(d)** Increase of water-rock ratio from 0.003 to 1 correlates with calculated alteration temperature increase from 147 to 283°C (colour scale indicates the degree of rock alteration) which is consistent with our thermal image datasets acquired in 2014 (Darmawan et al., 2018)^25^.

**Supplementary Table 1.** Description of Merapi lava dome samples collected for this study.

| 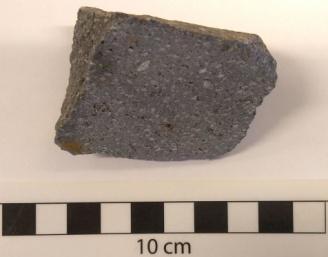 | The Fresh Merapi dome lava (MF) sample is light grey in colour but is slightly darker on weathered surfaces. It is porous and displays a porphyritic texture with plagioclase phenocrysts being visibly abundant. Black needle-like pyroxene crystals and small amounts of amphibole crystals are also present, but less abundant than plagioclase (with various sizes up to c. 5 mm). No cracks are visible and pores are mostly intact with no apparent stains in the specimen.  *Coordinates of sample: -7° 32' 21.5" S 110° 26' 51.5" E*  *Elevation: 2838 m a.s.l* |
| --- | --- |
| 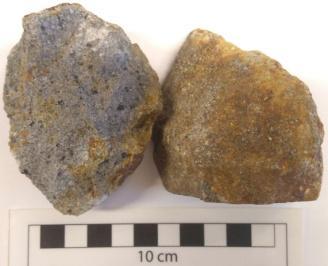 | The Slightly Altered Merapi dome lava (MSA) sample is light grey in colour and fresh surfaces are irregularly stained with tan white to pale red colour. The sample is porous and some pores have been filled with hydrothermal minerals. Relict plagioclase and pyroxenes are still present with sizes of up to several mm. Plagioclase is in part degraded in hand lense view and appears to have been partly replaced.  *Coordinates of sample: -7° 32' 23.1" S 110° 26' 51.2" E*  *Elevation: 2876 m a.s.l* |
| 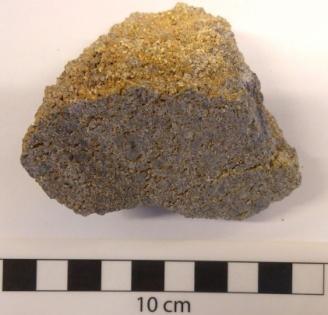 | The Moderately Altered Merapi dome lava (MMA) is grey coloured, moderately porous, and exposed surfaces and pores are stained by white to tan yellow colour from hydrothermal alteration minerals (e.g. alunite and gypsum). Primary magmatic minerals are hard to identify due to alteration, but some crystal boundaries are preserved and some bigger crystals are still visible.  *Coordinates of sample: -7° 32' 23.1" S 110° 26' 51.2" E*  *Elevation: 2882 m a.s.l* |
| 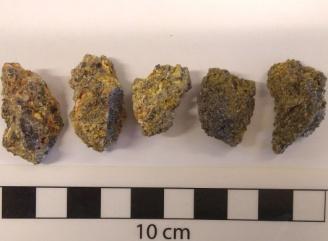 | The Highly Altered Merapi dome lava (MHA) is yellowish grey in colour, very porous and little of the original texture is preserved. Only few recognisable relics of pyroxene and feldspar remain and are not yet replaced by alunite. Sulphur-rich stains fill the pores and envelope remaining phenocryst. Red stains from hematite are visible in some parts.  *Coordinates of sample: -7° 32' 24" S 110° 26' 50.4" E*  *Elevation: 2905 m a.s.l* |
| 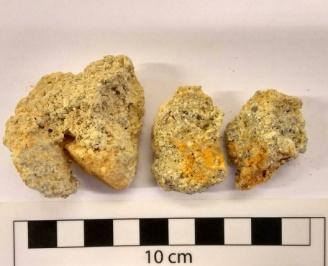 | The Fully Altered Merapi dome lava (MFA) is yellow in colour with orange stains. The original rock texture is not preserved. All minerals are completely altered and the groundmass is entirely replaced by sulphurous mineral assemblages. Primary magmatic crystals are no longer recognisable. The rock easily crumbles and is relatively low in density.  *Coordinates of sample: -7° 32' 24.1" S 110° 26' 50.8" E*  *Elevation: 2902 m a.s.l* |

**Supplementary Table 2.** Mineral changes in Merapi summit samples. Hydrothermal alteration of andesitic dome rock at Merapi produces up to 40% sulfate dominated minerals assemblage in the most altered specimens and significantly reduces the amount of primary feldspar and clinopyroxene.

| Rocks | Sample | Minerals (%) | | | | | | | | |  |
| --- | --- | --- | --- | --- | --- | --- | --- | --- | --- | --- | --- |
|  |  | Andes | Kfs | An | Cpx | Mt | Hm | Na-Alu | Gy | Jrs | δ^18^O (‰) |
| Fresh  Altered | MF-01 | 81 |  |  | 18.5 | 0.5 |  |  |  |  | 7.4 |
|  | MF-02 | 80.8 |  |  | 19 | 0.2 |  |  |  |  | 7.6 |
|  | MSA-01 | 57.2 | 29.6 |  | 13.1 |  |  |  |  |  | 8.3 |
|  | MSA-02 | 58.4 | 30.4 |  | 11.2 |  |  |  |  |  | 8.0 |
|  | MMA-01 | 76.3 |  |  | 11.3 |  | 0.9 | 11.6 |  |  | 10.8 |
|  | MMA-02 | 76.3 |  |  | 11.7 |  | 0.9 | 11.1 |  |  | 10.9 |
|  | MHA-01 | 54.2 |  |  | 9.7 |  |  | 29.3 | 6.8 |  | 11.8 |
|  | MHA-02 | 51.6 |  |  | 7.1 |  |  | 31.6 | 9.7 |  | 11.9 |
|  | MFA-01 |  |  | 63.4 | 12.7 |  |  | 11.5 |  | 12.4 | 12.8 |
|  | MFA-02 |  |  | 68.1 | 10.9 |  |  | 20.4 |  | 0.6 | 12.7 |
| Condensed fumarole H_2_O fluid sample from Merapi summit (Humaida et al., 2017) ^45^ | | | | | | | | | | | -14.8 |

Andes: Andesine, Cpx: Clinopyroxene, Mt: Magnetite, Kfs: Potassium feldspar, An: Anorthite, Hm: Hematite, Na-alu: Natroalunite, Gy: Gypsum, Jrs: Jarosite.

**Supplementary Table 3.** δ^18^O, water-rock ratio, dry bulk sample density, connected porosity, uniaxial compressive strength, and permeability for the rock samples measured for this study. Porosity and permeability data from ref. ^27^.

| **Sample** | **δ^18^O (‰)** | **Water-rock ratio** | **Dry bulk sample density (kg/m^3^)** | **Connected porosity (%)** | **Uniaxial compressive strength (MPa)** | **Permeability (m^2^)** |
| --- | --- | --- | --- | --- | --- | --- |
| MF01 | 7.4 | 0.026 | 2578.3 | 8.1 | 145.0 | 2.73 ⋅ 10^-17^ |
| MF02 | 7.2 | -0.015 | 2559.2 | 8.7 | 124.4 | 4.16 ⋅ 10^-17^ |
| MF03 | 7.6 | 0.057 | 2572.7 | 8.3 | 143.9 | 1.41 ⋅ 10^-16^ |
| MF04 | - | - | 2575.6 | 8.0 | 130.6 | 8.50 ⋅ 10^-17^ |
| MF05 | - | - | 2580.8 | 8.0 | 142.1 | 2.47 ⋅ 10^-17^ |
| MF06 | - | - | 2563.4 | 8.5 | 117.0 | 3.18 ⋅ 10^-16^ |
| MF07 | - | - | 2565.4 | 8.3 | 123.3 | 2.17 ⋅ 10^-17^ |
| MSA01 | 8.3 | 0.194 | 2473.7 | 8.6 | 108.4 | 3.18 ⋅ 10^-18^ |
| MSA02 | 8.0 | 0.132 | 2484.5 | 8.2 | 126.1 | 1.26 ⋅ 10^-17^ |
| MSA03 | 7.9 | 0.117 | 2472.7 | 8.7 | 118.6 | 4.00 ⋅ 10^-18^ |
| MSA04 | - | - | 2478.0 | 8.0 | 130.2 | 3.23 ⋅ 10^-18^ |
| MSA05 | - | - | 2502.3 | 7.9 | 137.8 | 5.84 ⋅ 10^-18^ |
| MSA06 | - | - | 2483.3 | 8.4 | 109.2 | 2.01 ⋅ 10^-17^ |
| MSA07 | - | - | 2504.5 | 7.7 | 141.3 | 6.51 ⋅ 10^-18^ |
| MMA01 | 10.8 | 0.689 | 2232.9 | 15.9 | 42.8 | 4.07 ⋅ 10^-15^ |
| MMA02 | 10.9 | 0.703 | 2231.9 | 15.2 | 48.4 | 4.86 ⋅ 10^-17^ |
| MMA03 | 11.2 | 0.778 | 2109.6 | 17.6 | 40.3 | 5.99 ⋅ 10^-16^ |
| MMA04 | - | - | 2292.5 | 15.4 | 53.0 | 7.81 ⋅ 10^-15^ |
| MMA05 | - | - | 2207.3 | 18.2 | 35.3 | 8.93 ⋅ 10^-14^ |
| MMA06 | - | - | 2251.2 | 14.4 | 54.5 | 1.54 ⋅ 10^-16^ |
| MMA07 | - | - | 2265.6 | 15.5 | 47.9 | 9.35 ⋅ 10^-15^ |
| MHA01 | 11.8 | 0.887 | 2134.4 | 18.5 | 79.0 | 8.94 ⋅ 10^-16^ |
| MHA02 | 11.9 | 0.911 | 2125.7 | 18.2 | 58.5 | 1.31 ⋅ 10^-14^ |
| MHA03 | 12.2 | 0.966 | 2109.1 | 19.2 | 41.3 | 8.71 ⋅ 10^-17^ |
| MHA04 | - | - | 2060.7 | 21.5 | 33.8 | 1.34 ⋅ 10^-14^ |
| MHA05 | - | - | 2013.2 | 23.3 | 26.3 | 1.75 ⋅ 10^-13^ |
| MHA06 | - | - | 2036.0 | 22.0 | 31.5 | 6.62 ⋅ 10^-15^ |
| MHA07 | - | - | 2108.0 | 18.8 | 74.8 | 7.16 ⋅ 10^-16^ |
| MFA01 | 12.8 | 1.077 | 2101.9 | 23.8 | 24.7 | 4.13 ⋅ 10^-12^ |
| MFA02 | 12.7 | 1.059 | 2121.2 | 23.0 | 26.1 | 3.61 ⋅ 10^-12^ |
| MFA03 | 12.7 | 1.06 | 2075.4 | 24.8 | 13.3 | 3.50 ⋅ 10^-12^ |
| MFA04 | - | - | 2128.3 | 22.7 | 18.3 | 4.02 ⋅ 10^-12^ |
| MFA05 | - | - | 2103.0 | 23.5 | 11.9 | 3.92 ⋅ 10^-12^ |
| MFA06 | - | - | 2085.5 | 24.0 | 11.1 | 2.59 ⋅ 10^-12^ |
| MFA07 | - | - | 2117.8 | 23.2 | 26.5 | 4.12 ⋅ 10^-12^ |
| M2006-1 | 6-7.5(cf. ^44^) | - | 2492.49 | 11.9 | 80.5 | 1.84 ⋅ 10^-14^ |
| M2006-2 | 6-7.5(cf. ^44^) | - | 2453.29 | 13.6 | 65.3 | 6.99 ⋅ 10^-14^ |
| M2006-3 | 6-7.5(cf. ^44^) | - | 2497.99 | 12.4 | 80.1 | 8.77 ⋅ 10^-15^ |
| M2006-4 | 6-7.5(cf. ^44^) | - | - | 14.1 | 56.7 | 2.14 ⋅ 10^-13^ |
| M2006-5 | 6-7.5(cf. ^44^) | - | - | 11.9 | 80.2 | - 1. ⋅ 10^-14^ |
